# Genetic variants of the HLA-G/LILRB1 ligand-receptor axis in donors or recipients are prognostic covariates for rejection after living kidney transplantation

**DOI:** 10.3389/fimmu.2025.1697839

**Published:** 2026-01-05

**Authors:** Julian Hölzenbein, Sabine Schramm, Falko M. Heinemann, Andreas Heinold, Anja Gäckler, Johanna Reinold, Benjamin Wilde, Yannik Busch, Nina Gruenen, Wolfgang Peter, Peter Alexander Horn, Oliver Witzke, Hana Rohn, Vera Rebmann

**Affiliations:** 1 Institute for Transfusion Medicine, University Hospital Essen, University Duisburg-Essen, Essen, Germany; 2 Department of Nephrology, University Hospital Essen, University Duisburg-Essen, Essen, Germany; 3 HLA-Laboratory, Stefan-Morsch-Foundation, Birkenfeld, Germany; 4 Department of Infectious Diseases, University Hospital Essen, University Duisburg-Essen, Essen, Germany

**Keywords:** *HLA-G* 3´UTR-1, *HLA-G* 3´UTR-2, *LILRB1*, ILT-2, kidney transplantation, acute cellular rejection, humoral rejection

## Abstract

**Background:**

HLA-G is a non-classical HLA class I molecule that promotes transplant tolerance. It engages the inhibitory receptor LILRB1 on immune effector cells, suppressing cytotoxic responses and inflammation, while promoting tolerogenic and regulatory immune phenotypes. Polymorphisms in the *HLA-G* 3′ untranslated region (3′UTR) modulate HLA-G expression levels, and *LILRB1* promoter variants influence receptor expression. The combined effect on kidney transplant (KTx) rejection has not been systematically studied.

**Methods:**

Living donor–recipient pairs undergoing KTx were genotyped for nine variants in the *HLA-G* 3′UTR region and two single nucleotide polymorphisms (SNPs) in the *LILRB1* promoter (PROMO) regions. Haplotypes were arranged for both loci. Clinical endpoints were biopsy-proven T cell-mediated rejection (TCMR) within one year and antibody-mediated rejection (AMR) within five years post-transplant.

**Results:**

Donor positivity for *HLA-G* 3′UTR-1 or UTR-2 or negative for UTR-3 haplotype were associated with a significantly higher risk of TCMR in both univariate or multivariate analyses. Recipients lacking the *LILRB1*-PROMO CG haplotype also had an increased TCMR risk. The combination of an *HLA-G* 3’UTR-2 positive donor with a *LILRB1*-PROMO CG haplotype negative recipient was found to be an independent predictor of TCMR. In contrast, HLA-G 3′UTR variants were not associated with AMR, while the presence of the recipient *LILRB1*-PROMO CG haplotype emerged as an independent AMR risk factor.

**Conclusions:**

Donor *HLA-G* 3’UTR and recipient *LILRB1*-PROMO haplotypes define a functional immunogenetic axis that differentially influence TCMR and AMR. These results support the clinical potential of HLA-G/LILRB1 genetic profiling to improve donor selection in living KTx and to guide the development of novel rejection therapies.

## 1 Introduction

Human leukocyte antigen G (HLA-G) is a non-classical HLA class I molecule with potent immunomodulatory functions (1–4). Unlike classical HLA class I antigens, HLA-G exhibits limited polymorphism, restricted tissue distribution, and multiple isoforms, including both membrane-bound and soluble variants (5). Under physiological conditions, HLA-G expression is predominantly restricted to immune-privileged sites, such as the maternal–fetal interface, where it contributes to immune tolerance (6, 7). In pathological contexts, including transplantation, infection, autoimmunity, and malignancy, HLA-G expression can be induced and contributes to immune evasion by suppressing the activation and effector functions of various immune cells (8–17).

The immunosuppressive activity of HLA-G is primarily mediated through its interaction with inhibitory receptors expressed on immune effector cells (18). One of the key receptors is immunoglobulin-like transcript 2 (ILT2), also referred to as LILRB1 or CD85j (19). LILRB1 is expressed on a broad spectrum of immune cells, including subsets of CD4+ and CD8+ T cells, natural killer (NK) cells, B cells, and myeloid antigen-presenting cells (APCs). Notably, LILRB1 expression also varies by cell type, with high levels on monocytes and B cells and intermediate to low levels on NK cells and T cells. Upon engagement with HLA-G, LILRB1 transmits inhibitory signals that suppress cytotoxic activity, reduce cytokine production, inhibit cell proliferation, and promote tolerogenic or regulatory immune phenotypes (18, 20–27). Although HLA-G is its primary ligand, LILRB1 also binds classical HLA class I molecules and HLA-F, suggesting a broader role in fine-tuning immune responses (28, 29).

The HLA-G-LILRB1 interaction serves as a critical immune checkpoint that is essential for peripheral immune tolerance (30, 31). The efficiency of this interaction depends on the expression levels of both HLA-G ligand and LILRB1 receptor. Polymorphisms in the 3′ untranslated region (3′UTR) of the HLA-G, including multiple single nucleotide polymorphisms (SNPs) and the well characterized 14 base pair insertion/deletion (14 bp Ins/Del), modulate mRNA stability, splicing, and microRNA targeting, collectively shaping HLA-G expression (32–36). Functional studies have shown that the 14 bp insertion is associated with reduced mRNA stability and lower soluble HLA-G (sHLA-G) levels, whereas the +3142G allele enhances miR-148a/miR-152 binding and downregulates expression. These variants form defined haplotypes with differential expression and consequently immunoregulatory potential (37). Consequently, UTR-1 and UTR-2 haplotypes are linked to higher sHLA-G expression, while UTR-3 is associated with lower levels. Similarly, LILRB1 expression is regulated by SNPs such as rs10416697C/G in the distal promoter and rs1004443G/A in the proximal promoter regions (38, 39). These SNPs may alter transcriptional activity and surface receptor density. The rs10416697G and rs1004443A alleles are associated with higher promoter activity and increased LILRB1 surface expression in certain immune effector cells.

In kidney transplantation (KTx), alloimmune recognition of donor antigens by recipient immune system is a principal cause of rejection (40). Rejection is classified as T cell-mediated rejection (TCMR), involving direct cytotoxic and inflammatory responses, or antibody-mediated rejection (AMR), characterized by donor-specific antibodies (DSAs), complement activation, and endothelial injury. TCMR involves infiltration of CD8+ cytotoxic T cells and CD4+ helper T cells, leading to tissue destruction and inflammation. In contrast, AMR results from B cells activation and DSA production. Those antibodies bind to donor HLA molecules, activating the classical complement cascade, and inducing microvascular inflammation. NK cells and monocytes contribute to antibody-dependent cellular cytotoxicity through Fc receptor engagement, amplifying endothelial injury. C4d deposition in peritubular capillaries is a diagnostic hallmark. While TCMR can often be managed by immunosuppressive therapy, AMR remains difficult to treat and is closely associated with chronic graft dysfunction (41, 42).

Given its central role in immune regulation, the HLA-G–LILRB1 axis is of significant interest in transplantation immunology. Increased HLA-G expression has been associated with reduced rejection and improved graft survival. This effect being likely mediated through suppression of both cellular and humoral immune responses. However, the clinical relevance of this pathway may depend on the genetic variability of both ligand and receptor. While previous studies have evaluated the individual contributions of *HLA-G* 3′UTR polymorphisms, the role of *LILRB1* promoter variants and the combined effect of donor *HLA-G* 3′UTR haplotypes and recipient *LILRB1* haplotypes have not been systematically investigated.

This study aims to focus on the joint impact of donor and recipient *HLA-G* 3′UTR haplotypes and *LILRB1* promoter (PROMO) SNPs (rs10416697 and rs1004443) on rejection risk following KTx. Understanding this genetic interplay may provide novel insights into the mechanisms of transplant tolerance and contribute to the development of genotype-informed strategies for risk assessment, therapeutic targeting, and long-term graft survival optimization.

## 2 Materials and methods

### 2.1 Study population and clinical data

This study included 293 living donor–recipient pairs who underwent living KTx at the University Hospital Essen, Germany, between 2005 and 2017 as part of the institutional living donor kidney transplant program. Clinical and demographic data for donors and recipients were obtained from institutional medical records and transplant databases (**Table 1**). Donors were predominantly under 60 years of age (82.6%) and 56% were female. Recipients were mostly male (61.1%) and under 60 years of age (89.4%). The median cold and warm ischemia times were 135 minutes (range: 45–407) and 19 minutes (range: 10–76), respectively. High immunological disparity was present, with >2 HLA-A, -B, or -DR mismatches in 87.4% of cases, sex mismatches in 66.9%, and ABO-incompatible transplantation in 14.7%. Pre-transplant panel-reactive antibodies were detected in 11.9% of recipients, while 13.0% developed post-transplant donor-specific anti-HLA antibodies (DSAs). All patients received standard post-transplant follow-up care. The primary clinical endpoints were biopsy-proven TCMR within the first 12 months and AMR, diagnosed within five years post-transplant. Rejection episodes were classified according to the Banff classification system applicable at the time of biopsy (40, 43–46), based on histopathological criteria and the presence of DSAs where relevant. All biopsies were performed based on clinical indication. Induction immunosuppression consisted of basiliximab (anti-IL-2 receptor CD25 monoclonal antibody), followed by maintenance therapy with a calcineurin inhibitor (tacrolimus or cyclosporine), corticosteroids, and an antiproliferative agent (mycophenolate mofetil or azathioprine), in line with institutional and national guidelines. Antithymocyte globulin (ATG) was administered in 4.8% of recipients. No significant association was observed between the etiology of primary kidney disease and the key clinical outcomes assessed in this study.

**Table 1:** Demographic and clinical characteristics of recipients and donors at baseline.

Donor (N=293)	Number
Sex (men/women)	128/165
Age (<60 years/>60 years)	242/51
Recipient (N=293)	
Sex (men/women)	179/114
Age (<60 years/>60 years)	262/31
KTx related parameters	
Cold ischemia time, Median (range); minutes	135 (45 – 407)
Warm ischemia time, Median (range); minutes	19 (10 – 76)
Mismatch situation (MM)	
HLA-A,-B, or -DR MM (<2MM/>2MM)	37/256
Sex MM (no/yes)	97/196
AB0iTx (no/yes)	250/43
Panel reactive antibody (PRA)	
PRA pre-KTX (yes/no)	35/258
Initial Immunosuppressive therapy	
ATG/Basiliximab based induction therapy	14/279
CNI-administration yes/no	288/5
MMF co-administration yes/no	253/40
Steroid co-administration yes/no	290/3
other	1/292

HLA, Human Leukocyte Antigen; ATG, antithymocyte globulin; CNI, calcineurin inhibitor; MMF, mycophenolate mofetil; KTx, kidney transplant, AB0iTx, AB0 incompatible transplantation.

All participants provided written informed consent. The study protocol was approved by the local ethics committee of the Medical Faculty of the University of Duisburg-Essen (approval number 12-5312-BO) and conducted in accordance with the Declaration of Helsinki.

### 2.2 *HLA-G 3*′UTR and *LILRB1* rs10416697/rs1004443 genotyping

Genomic DNA was extracted from peripheral blood leukocytes using the QIAamp DNA Blood Mini Kit (Qiagen, Hilden, Germany) in accordance with the manufacturer’s protocol. The 3′ untranslated region (3′UTR) of the *HLA-G* gene was amplified by polymerase chain reaction (PCR), and sequencing was performed as recently described (9, 15). Sequence chromatograms were evaluated using FinchTV software version 1.4.0 (Geospiza Inc.). The sequencing analysis covered 10 polymorphic positions in the *HLA-G* 3′UTR, including the 14 bp insertion/deletion (+2961) and SNPs at +3001 C/T, +3003 C/T, +3010 C/G, +3027 A/C, +3035 C/T, +3142 C/G, +3187 A/G, +3196 C/G, and +3227 A/G.

The *LILRB1* promoter (*LILRB1*-PROMO) variants of rs10416697C/G (47) and of rs1004443G/A were defined using TaqMan SNP Genotyping Assays (Thermo Fisher Scientific, Waltham, MA, USA) according to the manufacturer’s instructions. Genotyping was performed on a real-time PCR platform (Quant Studio 6 Real-Time-PCR-System, Applied Biosystems, Thermo Fisher Scientific), and allele calls were made by endpoint fluorescence analysis using standard software provided by Thermo Fisher.

Haplotype inference was conducted using PHASE version 2.1 with default parameters (15). Inferred haplotypes had posterior probabilities ranging from 0.98 to 1.0 and demonstrated consistency across 10 independent runs.

### 2.3 Statistics

Statistical analyses were performed using SPSS Statistics version 27 (IBM Corp., Armonk, NY, USA), GraphPad Prism version 10.0 (GraphPad Software, San Diego, CA, USA), R (Rstudio), or MedCalc^®^ Statistical Software version 23.2.1 (MedCalc Software Ltd, Ostend, Belgium; https://www.medcalc.org; 2025). Graphical abstract was performed using BioRender. Allelic and genotypic distributions were assessed for deviation from Hardy–Weinberg equilibrium using Haploview version 4.2 (Broad Institute, Cambridge, MA, USA), which was also used to construct linkage disequilibrium (LD) plots and haplotype blocks based on LILRB1 promoter (LILRB1-PROMO) SNPs. Associations between HLA-G 3′UTR haplotypes, LILRB1-PROMO haplotypes, and clinical endpoints (including biopsy-proven rejection) were evaluated using univariate analysis Fisher’s exact test. Kaplan-Meier survival analysis with log-rank tests were performed by survminer R package version 0.4.9 (https://CRAN.R-project.org/package=survminer). Multivariate analysis was conducted using Cox proportional hazards regression to identify independent predictors of rejection, adjusting for relevant clinical and transplant-related variables. A two-tailed p-value < 0.05 was considered statistically significant.

## 3 Results

### 3.1 Distribution of *HLA-G* 3′UTR haplotypes in living kidney transplant recipients and donors

*HLA-G* 3′UTR haplotypes were determined in 280 kidney transplant recipients and 279 living donors. Nine haplotypes with a frequency >1% were identified in both groups. *HLA-G* 3′UTR-1 and *HLA-G* 3′UTR-2 were the most prevalent, accounting for 36.8% and 31.9% of haplotypes in recipients and 32.8% and 30.1% in donors, respectively. Haplotype distributions did not differ significantly between groups, consistent with prior population data (see **Table 2**, (5)). However, homozygosity for the *HLA-G* 3′UTR-1 haplotype was significantly more frequent in kidney transplant recipients than in donors (p = 0.002, **Supplementary Table 1**). In recipients, *HLA-G* 3′UTR-1 homozygosity was associated with underlying primary diseases such as glomerulonephritis (see **Supplementary Data**).

**Table 2:** HLA-G 3’UTR haplotype frequencies of recipients (n=280) and donors (n=279).

Haplotype	Frequency recipient	Frequency donor	p_a_	OR (95% CI)	Frequency europe*
UTR-1	36.8	32.8	0.161	1.19 (0.93 - 1.53)	34.7
UTR-2	31.9	30.1	0.502	1.09 (0.84 - 1.40)	28.3
UTR-3	8.4	8.8	0.817	0.95 (0.63 - 1.44)	9.1
UTR-4	14.8	15.1	0.913	0.98 (0.71 - 1.36)	14.8
UTR-5	2.3	3.6	0.212	0.63 (0.30 - 1.28)	3.3
UTR-6	1.9	16	0.657	1.22 (0.53 - 3.07)	0.5
UTR-7	6.1	5.0	0.442	1.22 (0.73 - 2.02)	5.5
UTR-13	1.4	1.8	0.629	0.79 (0.32 – 2.04)	0.2
UTR-18	2.1	2.0	>0.999	1.06 (0.49 - 2.17)	3.3

Haplotype phasing was assessed by PHASE 2.1 software using default parameters. Only haplotypes with frequencies >1 % were listed. UTR – untranslated region. p_a_-values were calculated by GraphPad Prism using two-sided Fisher’s exact test when evaluating haplotypes frequencies genotypes; alpha<0.05; OR-odds ratio; CI -confidence interval; *:Literature: https://doi.org/10.1038/s41598-021-02106-4.

### 3.2 Distribution of *LILRB1* promoter SNP genotypes in living kidney transplant recipients and donors

Genotyping of two single nucleotide polymorphisms (SNPs) - rs10416697 and rs1004443 - located in the promoter regions of the *LILRB1* gene was performed in kidney transplant recipients and living donors (**Table 3**). For rs10416697, there was no significant difference in C carrier frequency (CC + CG: 54.5% in recipients vs. 53.4% in donors; p = 0.8027; OR = 1.05, 95% CI: 0.76–1.44) or G carrier frequency (CG + GG: 91.3% vs. 88.4%; p = 0.2733; OR = 1.38, 95% CI: 0.81–2.33) was observed. For rs1004443 neither G carrier status (GG + AG: 53.2% vs. 53.8%; p = 0.9333; OR = 0.98, 95% CI: 0.71–1.35) nor A-carrier status (AA + AG: 91.5% vs. 87.9%; p = 0.1704; OR = 1.48, 95% CI: 0.87–2.55) differed significantly between recipients and donors. Overall, the distribution of *LILRB1*-PROMO SNP allelic frequencies was similar in both cohorts as well as in the European population-base data set ( (38), see **Table 4**).

**Table 3:** Distribution of LILRB1 SNP rs10416697 and rs1004443 genotypes detected at the LILRB1 promoter region in recipient (n=286/n=284) and donors (n=292/n=290).

SNP	Genotypes	Recipient	%	Donors	%	pc	OR (95% CI)
rs10416697	CC	25	8.7	34	11.6		
	CG	131	45.8	122	41.8	0.4135	
	GG	130	45.5	136	46.6		
	**C pos**						
	CC +CG vs.	156	54.5	156	53.4	0.8027	1.05 (0.76 - 1.44)
	GG	130	45.5	136	46.6		
	**G pos**						
	CG + GG vs.	261	91.3	258	88.4	0.2733	1.38 (0.81 - 2.33)
	CC	25	8.7	34	11.6		
rs1004443	AA	133	46.8	134	46.2		
	AG	127	44.7	121	41.7	0.3435	
	GG	24	8.5	35	12.1		
	**G pos**						
	GG + AG vs.	151	53.2	156	53.8	0.9333	0.98 (0.71 - 1.35)
	AA	133	46.8	134	46.2		
	**A pos**						
	AA + AG vs.	260	91.5	255	87.9	0.1704	1.48 (0.87 - 2.55)
	GG	24	8.5	35	12.1		

Cpos, Positive for C allele. Gpos, Positive for G allele.Apos, Positive for A allele.

**Table 4:** Distribution of LILRB1 SNP rs10416697 and rs1004443 alleles detected at the LILRB1 promoter region in recipient (n=286/n=284) and donors (n=292/n=290).

SNP	Allele	Recipient %	Donors %	pc	OR (95% CI)	Frequency Europe
rs10416697	C	31.6	32.5	0.7531	0.96 (0.75 - 1.23)	30.0
	G	68.4	67.5			70.0
rs1004443	A	69.2	67.1	0.448	1.10 (0.86 - 1.42)	70.0
	G	30.8	32.9			30.0

### 3.3 *LILRB1* promoter haplotype distribution in living kidney transplant recipients and donors

Both *LILRB1*-PROMO SNPs rs10416697 and rs1004443 were in strong linkage disequilibrium. Haplotype inference revealed four haplotypes (**Table 5**), which are referred to here as LILRB1-PROMO haplotype(s). The GA haplotype was most frequent in both groups (68.2% in recipients vs. 66.8% in donors; p = 0.611; OR = 1.07, 95% CI: 0.83–1.36), followed by CG (30.8% vs. 32.4%; p = 0.560; OR = 0.93, 95% CI: 0.72–1.19). The CA and GG haplotypes were rare (<1% in both groups) and did not differ significantly (CA: p = 0.988; GG: p = 0.186).

**Table 5:** LILRB1 gene promoter rs10416697 and rs1004443 haplotype (LILRB1-PROMO) frequencies of recipients and donors.

rs10416697, rs1004443 Haplotype	Frequency recipient	Frequency donor	p_a_	OR (95% CI)
CA	0.2	0.2	0.988	1.01 (0.05 - 19.43)
CG	30.8	32.4	0.560	0.93 (0.72 - 1.19)
GA	68.2	66.8	0.611	1.07 (0.83 - 1.36)
GG	0.2	0.7	0.186	0.25 (0.02 - 1.53)

### 3.4 Donor *HLA-G* 3′UTR-1, -2, -3 haplotypes are associated with the incidence of TCMR in the first year post living-kidney transplantation

To evaluate the impact of donor *HLA-G* 3′UTR polymorphisms on early TCMR, the association between individual donor haplotypes and the incidence of biopsy-proven TCMR within the first 12 months following kidney transplantation was analyzed.

**Figure 1:**
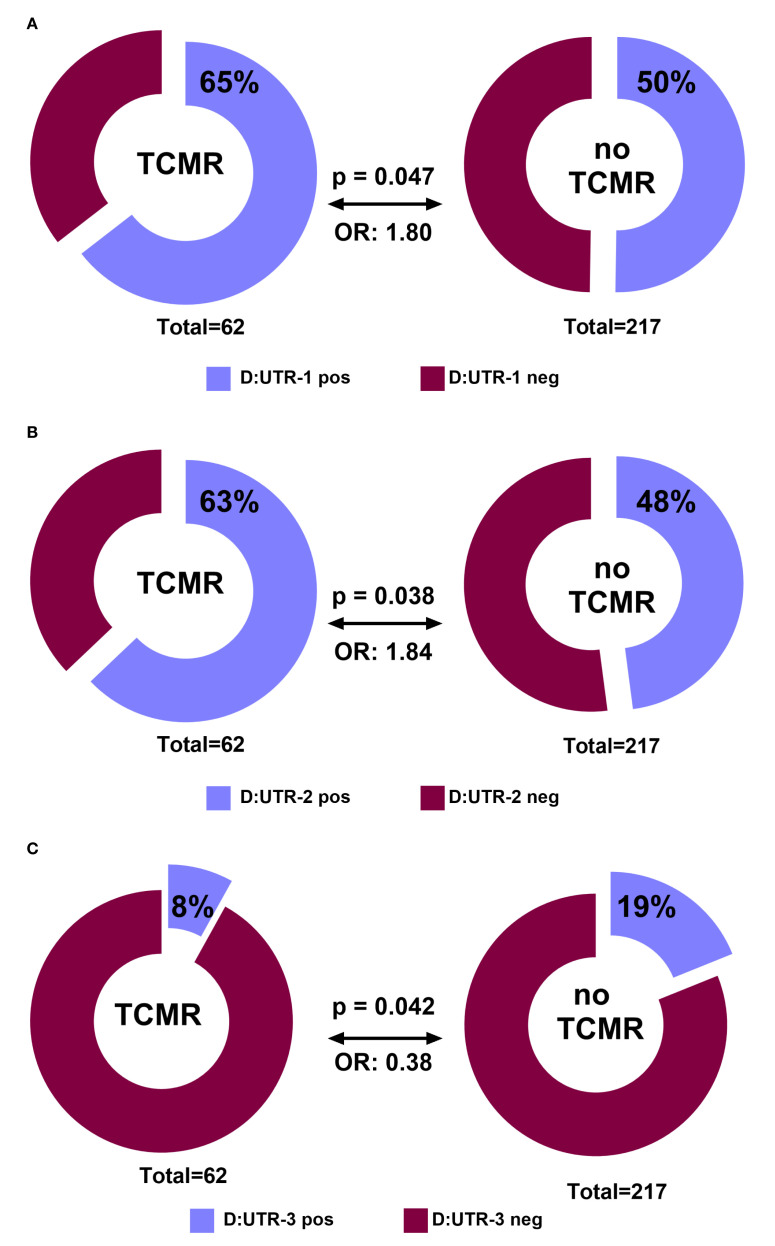
**(A–C)** Donor *HLA-G 3*′UTR haplotypes and their association with acute cellular rejection (TCMR) free survival within first year after KTx. Panels **(A–C)** show the frequency distribution of donor *HLA-G 3′*UTR-1, -2, and -3 haplotypes in the study cohort with the presence of TCMR within first year. Patient groups are distinguished by color codes as follows: Bordeaux (haplotype-negative) and violet (haplotype-positive) indicate the absence or presence of the respective haplotype.

Recipients of grafts positive for HLA-G 3′UTR-1 or 3′UTR-2 showed a higher likelihood of TCMR in univariate analysis (**Figures 1A–C**) (p = 0.047; OR = 1.80 and (p = 0.038; OR = 1.84, respectively). In contrast, donor *HLA-G* 3′UTR-3 carrier status was significantly associated with a lower incidence of TCMR compared to negative status (p = 0.042; OR = 0.38). No significant associations were observed for other *HLA-G* 3′UTR haplotypes from donors or recipients (data not shown).

**Figure 2:**
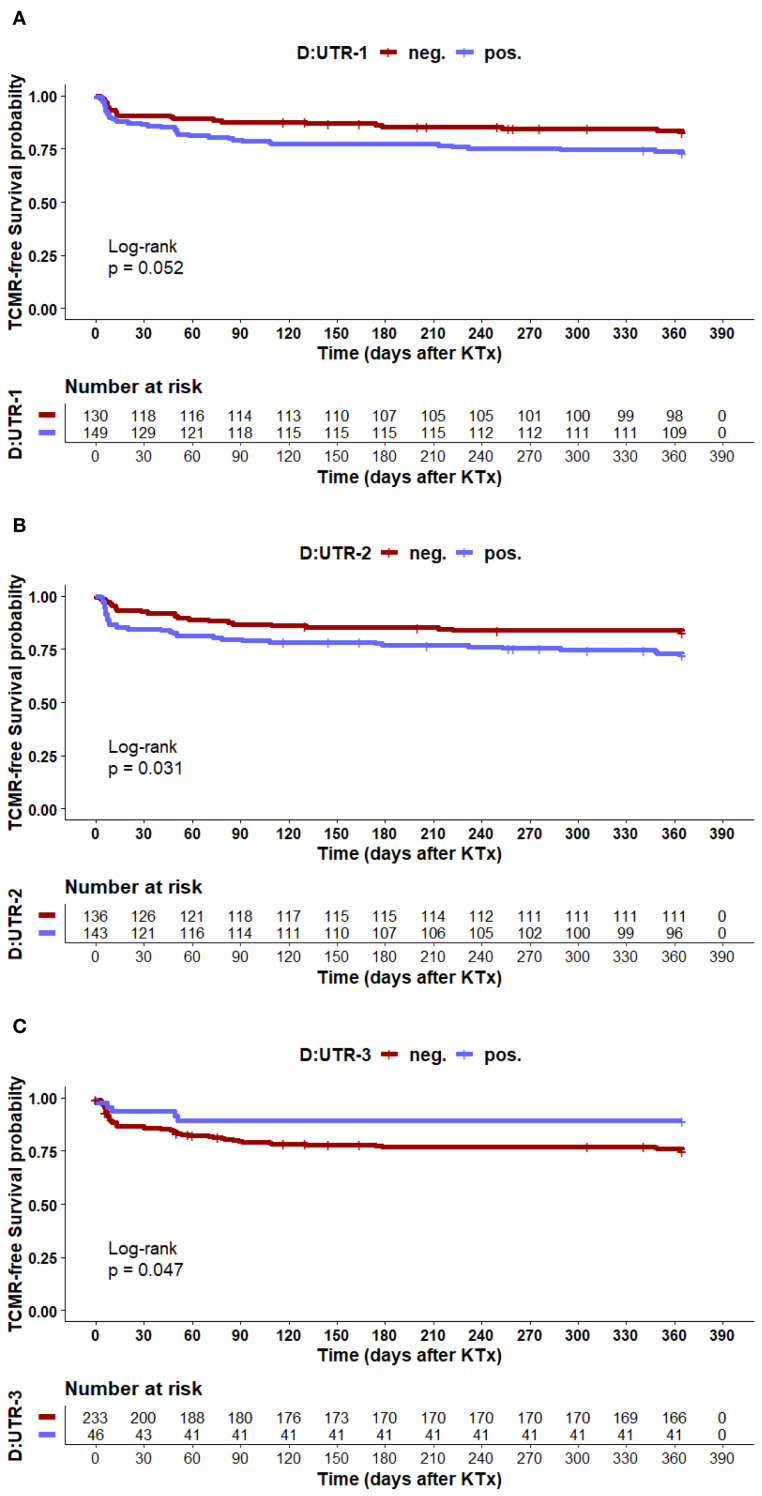
**(A–C)** The panels depict Kaplan–Meier plot analysis combined with log-rank for TCMR-free graft survival within the first 12 months after transplantation, stratified according to donor haplotype status **(D)** Graph A: HLA-G 3' UTR-1, Graph B: HLA-G 3'UTR-2 and Graph C: HLA-G 3'UTR-3. Tables under Kaplan–Meier plots show corresponding numbers at risk.

Taking into account the period of one year after KTx, these results were further corroborated by a Kaplan-Meier survival analysis with log-rank test (**Figures 2A–C**). A trend toward reduced TCMR-free survival probability was observed in *HLA-G* 3′UTR-1 haplotype-positive donor grafts, but statistical significance was not achieved (log-rank p = 0.052). Recipients who received a *HLA-G* 3′UTR-2-positive donor graft were associated with reduced TCMR-free survival (log-rank p = 0.031). In contrast, *HLA-G* 3′UTR-3-positive donor status was associated with prolonged TCMR-free survival probability (log-rank p = 0.047), suggesting a protective effect. Thus, donor *HLA-G* 3′UTR-1 and UTR-2 carrier status were related to greater TCMR risk, while *HLA-G* 3′UTR-3 was associated to protection in the univariate analysis.

### 3.5 Donor *HLA-G* 3′UTR-1 and *HLA-G* 3′UTR-2 haplotype status are independent predictors for TCMR post living-kidney transplantation in multivariate analysis

**Figure 3:**
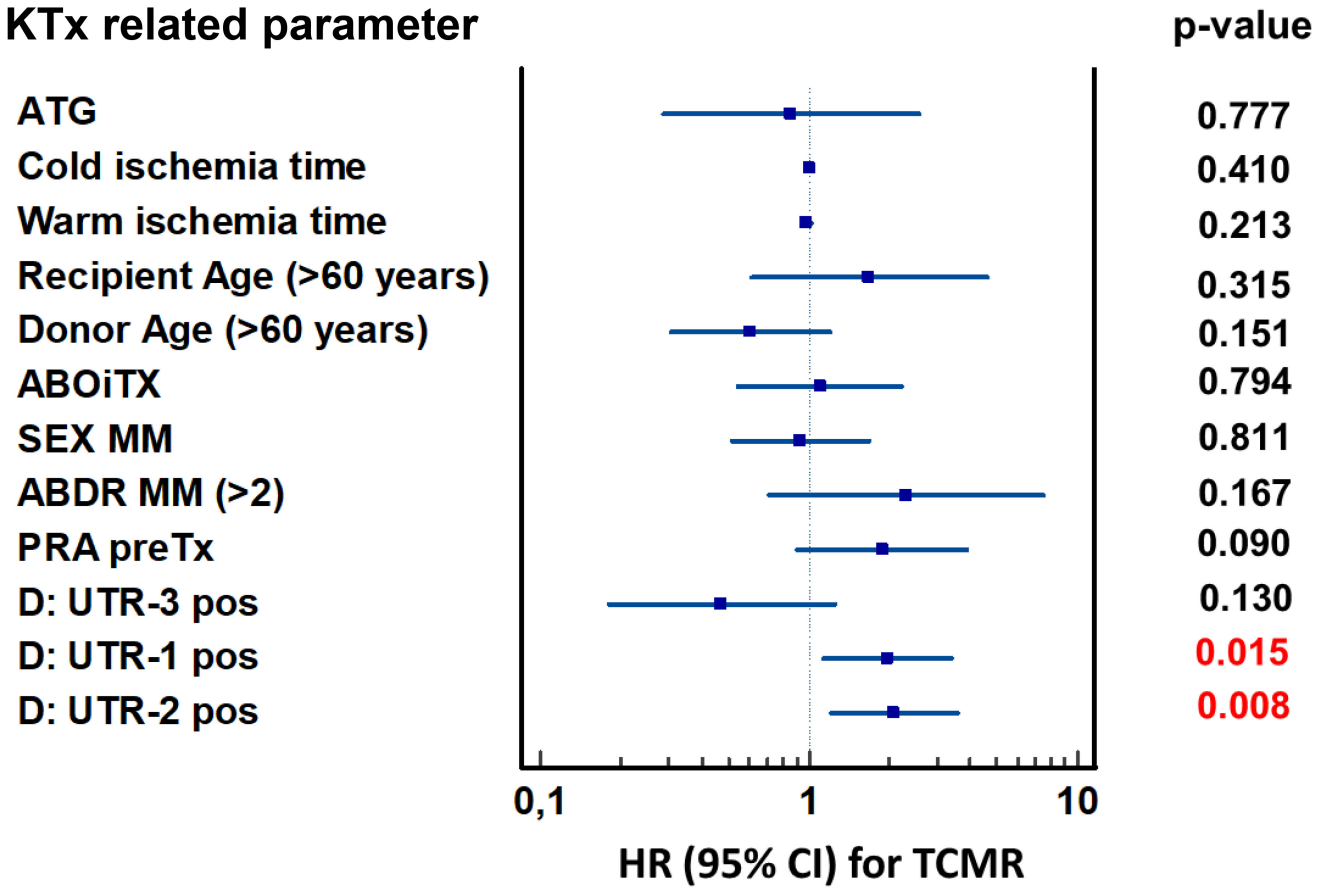
Forest plot of risk factors for TCMR within the first year after KTx. The forest plot shows the results of multivariate analyses for the following covariates: ATG induction, cold ischemia time, warm ischemia time, recipient age ≥ 60 years, donor age ≥ 60 years, ABO-incompatible transplantation, sex mismatch, >2 HLA-ABDR mismatches, pre-transplant PRA, and donor HLA-G 3′UTR-1, -2, and -3 carrier status. Donor *HLA-G 3*′UTR-1 and -2 haplotypes were associated with increased TCMR risk, while donor *HLA-G 3*′UTR-3 showed a protective effect. 95% CI, 95% confidence interval; HR, hazard ratio.

To assess the independent predictive value of donor *HLA-G* 3′UTR polymorphisms for TCMR, a multivariate Cox proportional hazards regression analysis was performed. The model was adjusted for transplant relevant clinical and immunological covariates, including ATG-based induction therapy, recipient and donor age, sex mismatch (MM), number of HLA-A, B, DR mismatch status (MM>2), ABO incompatibility (AB0i), panel-reactive antibody (PRA) status, cold and warm ischemia time. The forest plot illustrated the hazard ratios (HR) and 95% confidence intervals (CI) for each covariate (**Figure 3**). Among all variables tested, only the presence of donor *HLA-G* 3′UTR-1 (HR = 1.9, 95% CI: 1.1-3.4, p = 0.015) and *HLA-G* 3′UTR-2 (HR = 2.1, 95% CI: 1.2-3.6, p = 0.008) haplotypes were significantly associated with an increased risk of TCMR. In the Cox regression model, *HLA-G* 3′UTR-3 (HR: 0.47, 95% CI: 0.2-1.2, p = 0.130) did not reach the level of significance as an independent factor influencing TCMR risk. Furthermore, none of the clinical covariates included in the model were identified as independent prognostic covariates for TCMR. These results highlighted the independent and adverse impact of *HLA-G* 3′UTR haplotypes on TCMR.

### 3.6 Recipient *LILRB1* promoter CG haplotype status is a covariate for TCMR post living-kidney transplantation in univariate analysis

**Figure 4:**
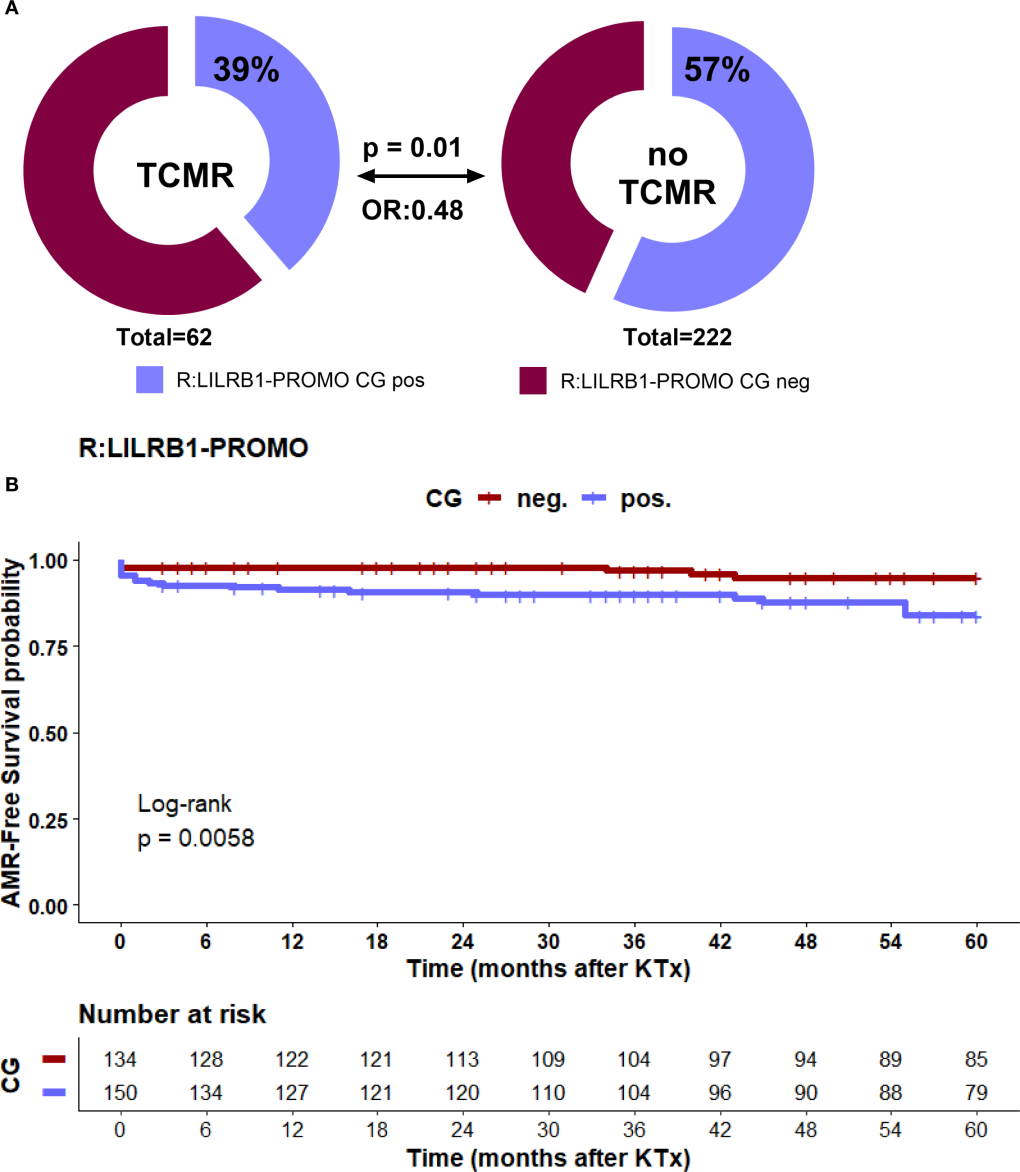
**(A, B)** Distribution of recipient *LILRB1*-PROMO CG haplotype and association with TCMR within first year after KTx. **(A)** The upper panel shows the frequency of the *LILRB1*-PROMO CG haplotype in kidney transplant recipients with the presence of TCMR within first year. Patient groups are distinguished by color codes as follows: Bordeaux (haplotype-negative) and violet (haplotype-positive) indicate the absence or presence of the respective haplotype. **(B)** Kaplan Meier plot analysis combined with log-rank for TCMR-free graft survival within the first 12 months after transplantation, stratified by recipient *LILRB1*-PROMO CG haplotype status. Recipients *LILRB1*-PROMO CG haplotype positive (violet) had significantly improved TCMR -free survival compared with *LILRB1*-PROMO CG haplotype negative recipients (bordeaux) (log-rank p = 0.011). Numbers at risk are shown below the survival curves.

To investigate the contribution of *LILRB1*-PROMO polymorphisms in modulating early alloimmune responses, we examined the association between the recipient *LILRB1*-PROMO haplotypes and the incidence of biopsy-proven TCMR within the first 12 months following kidney transplantation. Carriers of the LILRB1-PROMO CG haplotype exhibited a markedly lower incidence of TCMR (p = 0.01; OR: 0.48, **Figure 4A**). Consistently with this, Kaplan–Meier analysis with log-rank testing showed significant improved probability of 1-year TCMR-free survival in recipients carrying a *LILRB1*-PROMO CG haplotype compared to recipients lacking the *LILRB1*-PROMO CG haplotype (log-rank p = 0.011, **Figure 4B**). Importantly, no significant association was observed any donor *LILRB1*-PROMO haplotype and TCMR incidence (data not shown). Thus, these findings underscore the functional importance of *LILRB1* genetic variation in the promoter regions of the recipient in shaping the cellular alloimmune response following transplantation. The presence of *LILRB1*-PROMO CG haplotype confers a protective effect against TCMR.

### 3.7 The donor *HLA-G* 3′UTR-1/2 status and recipient LILRB1-PROMO haplotype status are independent covariates for TCMR post living-kidney transplantation in multivariate analysis

**Figure 5:**
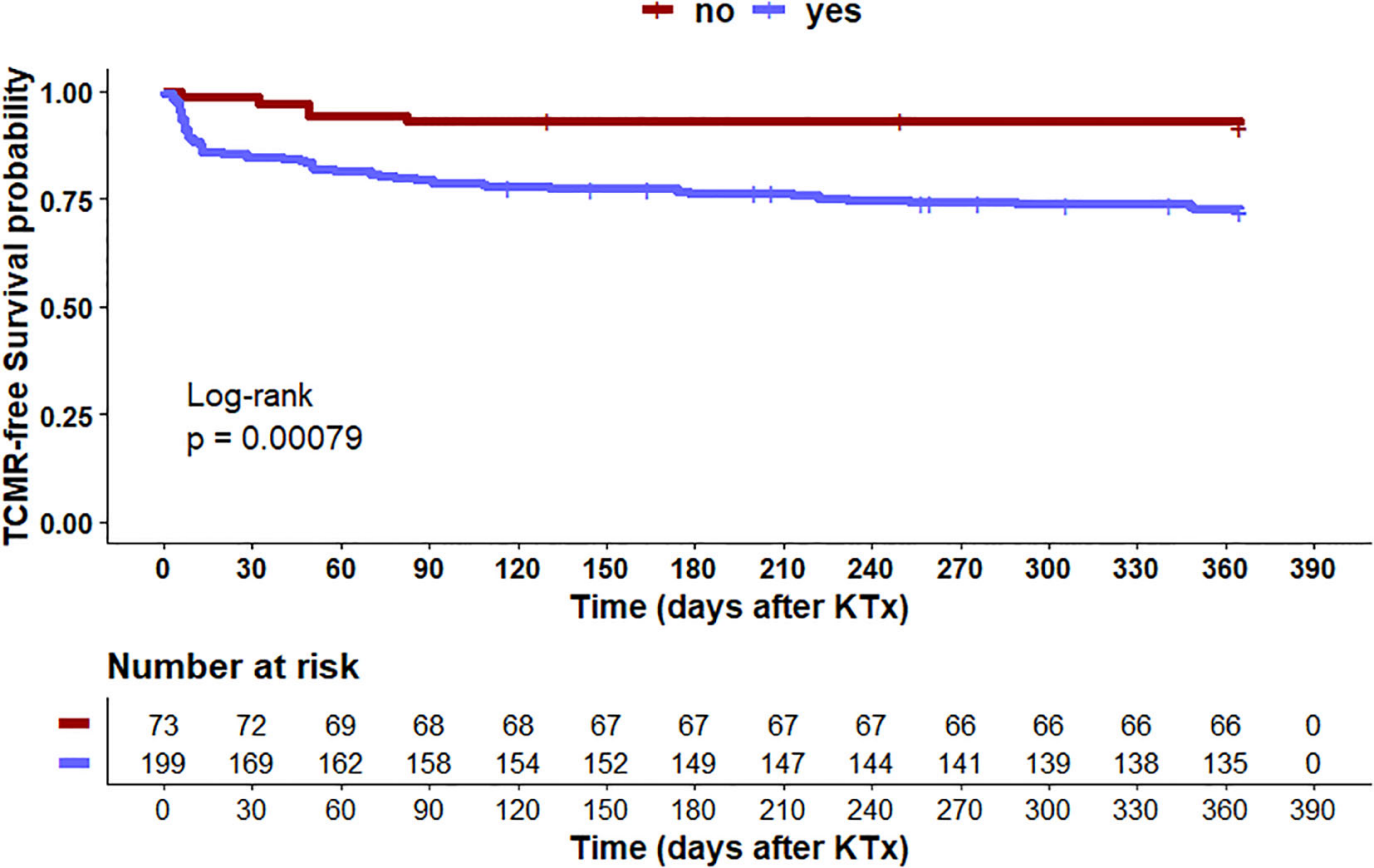
Forest plot of risk factors for TCMR within the first year after KTx. The forest plot shows the results of multivariate analyses for the following covariates: ATG induction, cold ischemia time, warm ischemia time, recipient age ≥ 60 years, donor age ≥ 60 years, ABO-incompatible transplantation, sex mismatch, >2 HLA-ABDR mismatches, pre-transplant PRA, donor HLA-G 3′UTR-1 and 3′UTR-2 haplotype status and recipient LILRB1-PROMO CG haplotype status. Donor HLA-G 3′UTR-1 and 3′UTR-2 were identified as independent risk factors for ACR, in addition to recipient LILRB1-PROMO CG haplotype negative status. 95% CI, 95% confidence interval; HR, hazard ratio.

To assess the individual contributions of donor *HLA-G* and recipient *LILRB1*-PROMO polymorphisms to the risk of TCMR), a multivariate Cox proportional hazards regression analysis was performed using clinical variables, consistent with those used in the evaluation of *HLA-G* 3′UTR haplotypes (**Figure 5**). The analysis demonstrated that donors positive for *HLA-G* 3′UTR-1 (HR = 1.9, 95% CI: 1.1-3.3, p = 0.023) or *HLA-G* 3′UTR-2 (HR = 2.0, 95% CI: 1.2-3.6, p = 0.011) haplotype had an significantly increased risk of TCMR within the first 12 months following transplantation. Additionally, the absence of *LILRB1*-PROMO CG haplotype in the recipient was independently associated with an increased risk of TCMR (HR = 1.48, 95% CI: 1.1-3.1, p = 0.021). No other genetic or clinical covariates reached statistical significance in the multivariate model.

### 3.8 The combination of an *HLA-G* 3′UTR-2-positive donor transplant and a *LILRB1*-PROMO CG haplotype-negative recipient is the strongest covariate for TCMR post living-kidney transplantation in the multivariate analysis

**Figure 6:**
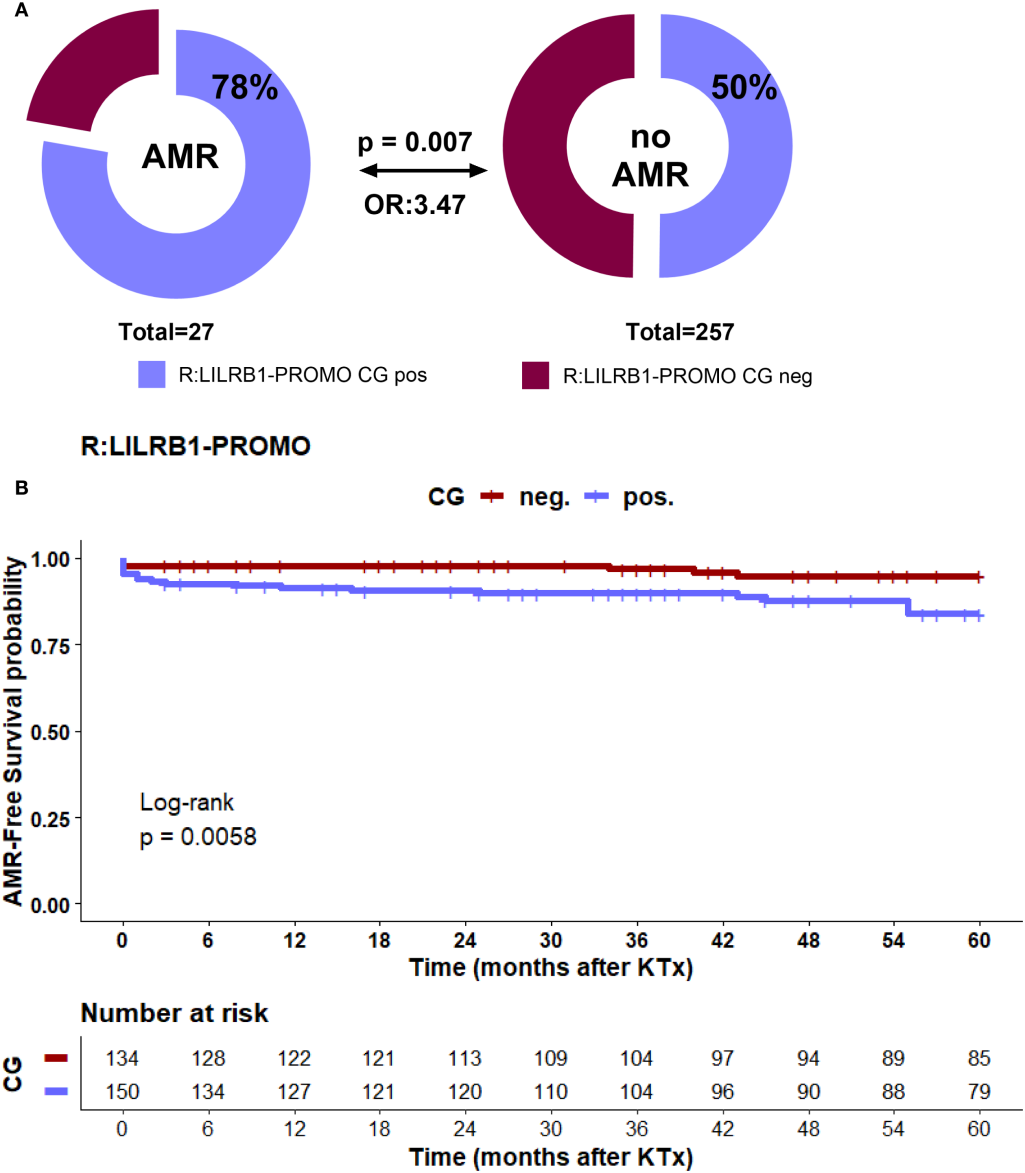
**(A, B)** Combined effect of donor HLA-G 3′UTR haplotypes and recipient LILRB1-PROMO CG haplotype on TCMR. **(A)** The forest plot shows the results of multivariate analyses for the following covariates: ATG induction, cold ischemia time, warm ischemia time, recipient age ≥ 60 years, donor age ≥ 60 years, ABO-incompatible transplantation, sex mismatch, >2 HLA-ABDR mismatches, pre-transplant PRA, and immunogenetic TCMR high risk constellations defined by donor HLA-G 3′UTR-1 or 3′UTR-2 positive status in combination with recipient LILRB1-PROMO CG negative status. Among these covariates, only donor HLA-G 3′UTR-2 positive status together with recipient LILRB1-PROMO CG negative status was identified as an independent risk factor for TCMR within the first year after KTx. In addition, >2 HLA-ABDR mismatches showed a borderline association with TCMR risk. 95% CI, 95% confidence interval; HR, hazard ratio. **(B)** Kaplan Meier plot analysis combined with log-rank for TCMR -free graft survival within the first 12 months after transplantation, stratified by combined donor–recipient immunogenetic status: Recipients of grafts from donors positive for HLA-G 3′UTR-2 and negative for the recipient LILRB1-PROMO CG haplotype had the lowest TCMR -free survival, representing the only constellation significantly associated with increased TCMR risk (log-rank p = 0.0058). In contrast, all other donor–recipient combinations showed superior TCMR -free survival. Numbers at risk are shown below the survival curves.

Since both donor and recipient haplotypes independently influence the risk of TCMR, and given the immunological significance of the HLA-G/LILRB1 checkpoint axis, an additional multivariate analysis was performed to assess whether specific donor-recipient haplotype combinations influence the occurrence of TCMR within the first year after transplantation. For this purpose, a high-risk combination was defined consisting of recipients lacking the *LILRB1*-PROMO-CG haplotype and donors carrying either the *HLA-G* 3′UTR-1 or *HLA-G* 3′UTR-2 haplotype (**Figure 6A**). The multivariate Cox regression analysis included the same transplant-related covariates as before. In this model, the only independent predictive TCMR risk factor was the immunogenetic pairing of recipients without the *LILRB1*-PROMO CG haplotype with donor transplants that were positive for *HLA-G* 3′UTR-2 (HR = 3.6, 95% CI: 1.6–8.5, p = 0.003). The pairing of *LILRB1*-PROMO CG negative-haplotype recipients with grafts positive for *HLA-G* 3′UTR-1 did not reach statistical significance in the multivariate analysis (HR = 1.7, 95% CI: 0.9–3.7, p = 0.12). None of the clinical covariates reached statistical significance. Accordingly, Kaplan-Meier survival analysis showed a significantly reduced probability of TCMR -free 1-year survival in recipients with a negative *LILRB1*-PROMO CG haplotype who received *HLA-G* 3′UTR-2-positive transplants compared to the other genetic combinations of donors and recipients (Log-rank p = 0.00079, **Figure 6B**).

### 3.9 Recipient *LILRB1*-PROMO CG positive-haplotype as a covariate for AMR post living-kidney transplantation in uni- and multivariate analysis

**Figure 7:**
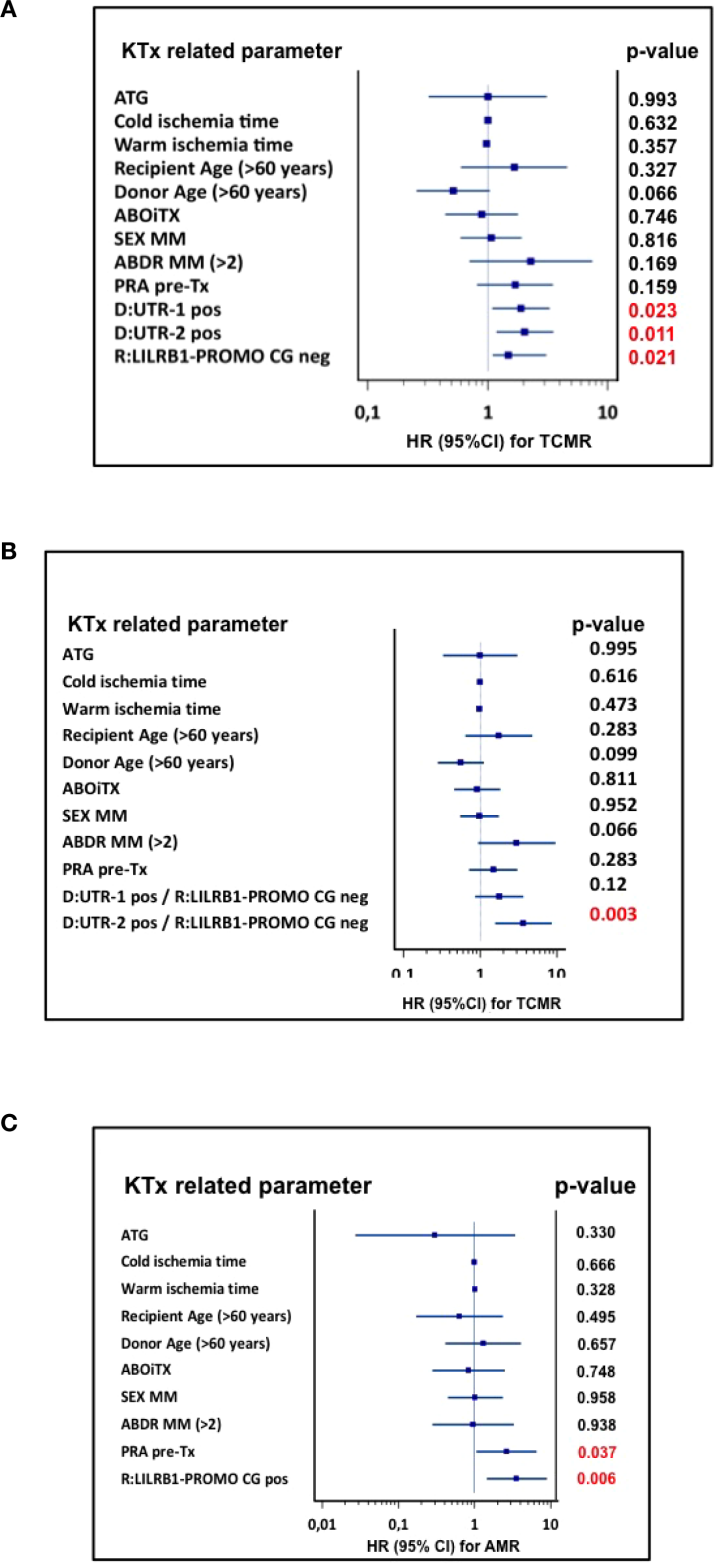
**(A–C)** Recipient LILRB1-PROMO CG haplotype and risk of AMR within 5 years after KTx. **(A)** Frequency of the LILRB1-PROMO CG haplotype in kidney transplant recipients regarding AMR. Patient groups are distinguished by color codes as follows: Bordeaux (haplotype-negative) and violet (haplotype-positive) indicate the absence or presence of the respective haplotype. **(B)** Kaplan–Meier plot analysis combined with log-rank test for AMR -free graft survival within 5 years after transplantation, stratified by recipient LILRB1-PROMO CG haplotype status. Recipients positive for the LILRB1-PROMO CG haplotype (violet) had significantly reduced AMR -free survival compared with haplotype-negative recipients (bordeaux) (log-rank p = 0.0058). Numbers at risk are shown below the survival curves. **(C)** Forest plot shows the results of multivariate analyses for the following covariates: ATG induction, cold ischemia time, warm ischemia time, recipient age ≥ 60 years, donor age ≥ 60 years, ABO-incompatible transplantation, sex mismatch, >2 HLA-ABDR mismatches, pre-transplant PRA, and recipient LILRB1-PROMO CG haplotype positivity. Recipient LILRB1-PROMO CG positivity was identified as an independent risk factor for AMR as well as pretransplant PRA status. 95% CI, 95% confidence interval; HR, hazard ratio.

To evaluate the impact of genetic and clinical parameters on the risk of AMR a comprehensive analysis was performed. Neither the donor’s nor the recipient’s *HLA-G* 3′UTR haplotypes showed a significant association with the occurrence of AMR during a five-year observation period. In contrast to TCMR, where the presence of the *LILRB1*-PROMO CG haplotype in recipients has a protective effect against rejection post living-kidney transplantation (**Figures 4, A, B**), this haplotype was significantly more common in patients with AMR than in patients without this *LILRB1*-PROMO haplotype (78% vs. 50%, p = 0.007, **Figure 7A**). Kaplan–Meier survival analysis further supported this finding, showing significantly reduced AMR -free survival probability in recipients carrying *LILRB1-*PROMO CG haplotype compared to non-carriers (log-rank p = 0.0058, **Figure 7B**). Importantly, the multivariate Cox proportional hazards model including the clinical covariates as before, demonstrated the *LILRB1*-PROMO CG haplotype carrier status (HR = 2.6, 95% CI: 1.1-6.5, p = 0.003) in addition to pre-transplant panel-reactive antibody positivity as independent predictors for AMR (HR = 3.5, 95% CI: 1.4-8.8, p = 0.037, **Figure 7C**). The latter finding has been previously described as an independent immunological risk factor in other kidney transplant cohorts. No other clinical variables demonstrated significant associations.

## 4 Discussion

Living donor kidney transplantation remains the best available therapeutic option for patients with end-stage renal disease, offering superior long-term graft survival, reduced waiting times, and lower rates of delayed graft function compared to deceased donor transplantation (48, 49). Immune-mediated rejection, however, remains a major cause of graft loss. Identifying genetic factors that influence rejection risk is critical for improving transplant outcomes. In our study donor *HLA-G* 3′UTR haplotypes and recipient *LILRB1* (PROMO) promoter polymorphisms (rs10416697 and rs1004443) were identified as immunogenetic relevant determinants of rejection risk following living kidney transplantation. Distinct donor–recipient haplotype constellations within the HLA-G/LILRB1 immune checkpoint axis differentially influence the incidence TCMR, whereas exclusively the recipient *LILRB1*-PROMO CG haplotype influences AMR. This suggests fundamentally different immune mechanisms underlying these two types of allograft injury.

HLA-G and its cognate LILRB1 receptor play a pivotal role in modulating alloimmune responses after transplantation (8, 50). This pathway induces induction of tolerogenic dendritic cells and regulatory T cells, impairment the effector cell function of T-, NK- and B- cells, thus contributing to both local and systemic immune modulation (51). The expression of both receptor and ligand is tightly regulated by genetic polymorphisms (39, 52).

HLA-G, unlike classical HLA class I molecules, is selectively expressed in immune-tolerant environments such as the placenta, tumors, and transplanted tissues and is tightly regulated at the transcriptional and post-transcriptional levels (36, 53). Functional variants in the 3′UTR influence HLA-G expression by altering mRNA stability, microRNA binding, and isoform expression. Six microRNAs (miR-148a, miR-148b, miR-152, miR-133a, miR-628-5p and miR-548q) have been shown to bind sequence specifically to certain SNPs within the HLA-G 3′UTR, thereby down-regulating its expression levels (54, 55). Their regulatory activity appears tissue-dependent (11, 56, 57), and many have been detected in renal cell carcinoma and other renal diseases (58, 59). Upon KTx, effect of immunosuppression on miRNA levels has so far not been clarified. The polymorphisms in the 3´UTR region arranged into nine major haplotypes, with HLA-G 3′UTR-1 and -2 being the most common (37, 60). These haplotypes have been associated with differential soluble (s)HLA-G expression. *HLA-G* 3′UTR-1 was associated with increased sHLA-G levels, whereas *HLA-G* 3′UTR-2 has low to intermediate HLA-G levels (32). Of note, only soluble HLA-G dimers - especially HLA-G5 - are reported to effectively transmit inhibitory signals via LILRB1 to suppress cytotoxic CD8^+^ T cell activity by downregulating Granzyme (2, 61–63). In line with this, increase sHLA-G expression has been associated with improved graft outcomes in different transplant settings (21, 50, 64–67).

The analysis of both *HLA-G* 3′UTR donor -recipient haplotypes in transplantation is scarce (9). In contrast to our study analysing matched recipient and the donor pairs, the majority of studies focus solely on recipient *HLA-G* 3′UTR polymorphisms. So far in lung transplantation certain *HLA-G* 3′UTR recipient haplotypes have been associated with impaired graft outcomes (68). In contrast to *HLA-G* 3′UTR haplotypes, defined SNPs of the *HLA-G* 3′UTR region are discussed to be associated with graft rejection or survival: particularly the 14-bp ins/ins genotype and the +3142GG variant, have been associated with a reduced risk of rejection (69–75). In contrast, the +3196G allele has been linked to an increased risk of graft loss (69). In our previous study the *HLA-G* 3´UTR-4 in both donor and recipient has been linked to an increased risk of BK polyomavirus (BKPyV) nephropathy and CMV replication following transplantation (9). Both infections are well known to be detrimental in transplant recipients, as BKPyVAN is a major cause of allograft dysfunction and loss, while CMV contributes to morbidity, mortality, and graft injury in kidney transplant recipients (76–78). Furthermore, the donor *HLA-G* 3′UTR-2 was already related with higher KTx rejection rates in univariate analysis (9). In the current expanded transplant cohort, this association between donor *HLA-G* 3´UTR-2 and acute rejection could be confirmed in univariate and in addition in multivariate analyses. Moreover, in the present study, the donor *HLA-G* 3′UTR-1 also emerged as a risk factor for TCMR in both univariate and multivariate analyses, whereas the donor *HLA-G* 3′UTR-3 seemed to exhibit a protective effect against TCMR. However, this association was only significant in univariate analysis and not in multivariate analysis. No associations were observed between recipient HLA-G 3′UTR haplotypes and TCMR, and no significant associations were found between donor or recipient *HLA-G* 3′UTR haplotypes and AMR, emphasizing the regulatory complexity of the *HLA-G* 3′UTR region in different pathological situations. Thus, our findings suggest that the predominant effect likely arises from locally produced HLA-G within the graft microenvironment rather than from systemic recipient levels.

Interestingly, we found that *HLA-G* 3′UTR-1 homozygosity was associated with underlying primary diseases such as glomerulonephritis, a group of primary diseases for which genetic predisposition has been implicated. This finding is consistent with previous studies linking *HLA-G* 3′UTR-1 to other diseases with a known genetic predisposition (14). However, regarding the primary outcomes, namely the occurrence of rejection episodes there was no association with the underlying primary diseases in our study cohort (data not shown).

HLA-G modulates local and systemic immune tolerance by engaging inhibitory receptors, primarily LILRB1 and LILRB2 (ILT4), on lymphoid and myeloid cells, thereby suppressing cytotoxicity and inflammation while promoting tolerogenic dendritic cells and regulatory T cells (63, 79–82). Thus, impaired LILRB1 function or expression has been implicated in the pathogenesis of several autoimmune diseases (83, 84). While LILRB2 has a high-affinity receptor for HLA-G (85), LILRB1 predominantly interacts with the β2m-associated HLA-G isoforms that are more abundantly expressed *in vivo*. Given its broader expression on both lymphoid and myeloid cells, LILRB1 is therefore more likely than LILRB2 to mediate HLA-G–dependent immune regulation in transplantation contexts.

Emerging evidence suggests that LILRB1 expression is modulated by polymorphisms in its promoter region, particularly rs10416697 and rs1004443 variants. These variants are believed to influence transcription factor binding and promoter activity, ultimately affecting LILRB1 surface expression on immune cells. The *LILRB1*-PROMO CG haplotype has been associated with a reduced frequency of NK cells expressing this receptor (71). However, this variation does not affect receptor expression on myeloid effector cells, which constitutively express LILRB1. So far, data regarding LILRB1 expression on T cells was not established. Of note, although LILRB1 is often expressed on T cells, its expression is heterogeneous across CD4+ and CD8+ T cell subsets, with only a small subpopulation of especially CD8+ T cells displaying LILRB1 on their surface. This highlights the complex regulation of HLA-G–LILRB1 interactions.

In has been demonstrated that elevated sHLA-G levels or the presence of extracellular vesicles containing HLA-G increase the frequency of CD8+ LILRB1+ T cells (27). Moreover, an increase of LILRB1 mRNA expression was reported in NK and T cells under the influence of elevated sHLA-G levels (86). With respect to transplantation setting, there are no data available how LILRB1 regulation and its expression are altered under the influence of immunosuppressive medication.

In our study, the analysis of donor and recipient *LILRB1*-PROMO haplotype revealed exclusively the importance of the presence of recipient *LILRB1*-PROMO CG haplotype to be protective for TCMR, whereas it was an independent risk factor of AMR besides the well-known recipient PRA-positivity. Recent findings underscore the immunological relevance of LILRB1 in the context of kidney transplant rejection. LILRB1 expression is elevated in circulating monocytes of kidney transplant recipients. Notably, myeloid cells isolated from kidney biopsy specimens show up-regulation of LILRB1, LILRB2 and LILRB3 following AMR, highlighting the involvement of this receptor family in the immune response to allografts (87). In line with this, single-cell transcriptomic profiling has demonstrated that recipient-derived monocytes expressing LILRB1 infiltrate the allograft during rejection episodes (87). These data indicate a functional role for LILRB1-expressing myeloid cells in mediating alloresponses. Collectively, these findings highlight LILRB1 as a key immune checkpoint receptor and support its potential as a target for modulating allo-immune activation and impacting transplant outcomes.

LILRB1 is not limited to binding HLA-G, it also recognizes classical HLA class I molecules (HLA-A, -B, -C) and HLA-F but also ligands that derive from various pathogens (especially Cytomegalovirus UL18 and E. coli) (88, 89). These alternative ligands, whose expression may be unregulated during inflammation or rejection, can engage LILRB1 in a conformation-dependent manner, potentially modulating immune outcomes in a context-specific fashion. Beyond ligand diversity, recent studies have highlighted that engagement of the CD47–SIRPα axis can influence the expression of leukocyte Ig-like receptors on myeloid cells, suggesting additional layers of regulation that may intersect with HLA-G/LILRB1 signaling pathways (90, 91). In addition, LILRB1 has also been linked to metabolic pathways, including cholesterol homeostasis and ferroptosis resistance in malignancies such as multiple myeloma, underscoring its functional relevance beyond transplant immunology and highlighting its potential as a target for novel therapeutic strategies (92).

While enhanced LILRB1 expression may support a more tolerogenic or inhibitory profile in antigen-presenting cells, which could suppress early T cell priming and mitigate TCMR, the same polymorphic variant may also influence B cell signaling, differentiation, or survival—thereby facilitating humoral alloimmunity (93). Indeed, LILRB1 signaling on B cells has been shown to impact their responsiveness and function, particularly in inflammatory or alloantigen-rich environments (94). In view of this context LILRB1 promoter haplotype was only important for AMR when present in the recipient, whereas the HLAG 3´UTR of the donor had no impact on AMR rate.

Taken together, our results suggest that the protective effect of HLA-G-LILRB1 interactions in TCMR is primarily driven by donor-recipient specific genetic determinants, whereas in AMR the recipient LILRB1 genetic variants have the most prominent effect. Thus, the HLA-G/LILRB1 axis is an important immune checkpoint not only in tumor biology but also in transplant settings. Interestingly in triple negative breast cancer the *HLA-G* 3´UTR haplotypes was not associated with disease outcome, however, high levels of sHLA-G combined with the *LILRB1*-PROMO rs10416697C allele being part of the here defined *LILRB1*-PROMO CG haplotype, were associated in adverse disease outcome in uni- and multivariate analysis (47).

These findings are clinically significant for enhancing pre-transplant risk stratification, particularly in living donor kidney transplantation cohort, which is the focus of this study. Our data suggest that specific HLA-G and LILRB1 genetic variants may differentially influence rejection risks. Specific donor *HLA-G* haplotypes and recipient *LILRB1*-PROMO variants not only influence the risk of rejection independently, but also interact functionally to define graft tolerance or injury. Our results support the integration of HLA-G and LILRB1 genotyping into routine pre-transplant immunogenetic assessment and highlight their potential utility in guiding individualized immunosuppressive management (95). Furthermore, our study is also applicable with regard to therapeutical approaches in terms of using sHLA-G or HLA-G positive extracellular vesicles as modulator of immune responses especially in the difficult to treat situation of AMR (94, 96–99). In the setting of stem cell transplantation, the application of HLA-G positive extracellular vesicles drastically improved therapy-refractory graft-versus-host disease (100).

Nevertheless, this study has several limitations making further studies necessary: i) the functional impact of *HLA-G* 3′UTR haplotypes remains context- and microenvironment-dependent, and is still poorly understood since no mRNA or protein levels were analyzed, ii) the sample size and exclusive focus on living-donor kidney transplantation from one transplant center in Germany may limit the generalizability of the findings to other transplant settings, iii) LILRB1 expression on T cell subsets and other immune effector cells in the donor organ as well as in the recipient periphery is insufficiently characterized, yet may be critical for understanding its role in rejection and tolerance induction and iv) our study did not include direct functional validations.

In conclusion, this study underscores the importance of the donor HLA-G and recipient LILRB1 immune checkpoint axis in shaping transplant outcomes. The results of our study support the potential utility of the HLA-G/LILRB1 immune checkpoint axis. Integrating HLA-G/LILRB1 genotyping into pre-transplant assessment could enhance donor–recipient matching and guide personalized immunosuppressive strategies. Future work should explore therapeutic modulation of this pathway, including the potential use of sHLA-G or HLA-G–positive extracellular vesicles to promote immune regulation and improve long-term graft survival.

## Data Availability

The original contributions presented in the study are included in the article/**Supplementary Material**. Further inquiries can be directed to the corresponding authors.
