# Supplementary figures and images for: Genetic variants of the HLA-G/LILRB1 ligand-receptor axis in donors or recipients are prognostic covariates for rejection after living kidney transplantation
Source: Front Immunol. 2026 Jan 5;16:1697839. doi: 10.3389/fimmu.2025.1697839 (PMC12812672; doi:10.3389/fimmu.2025.1697839)

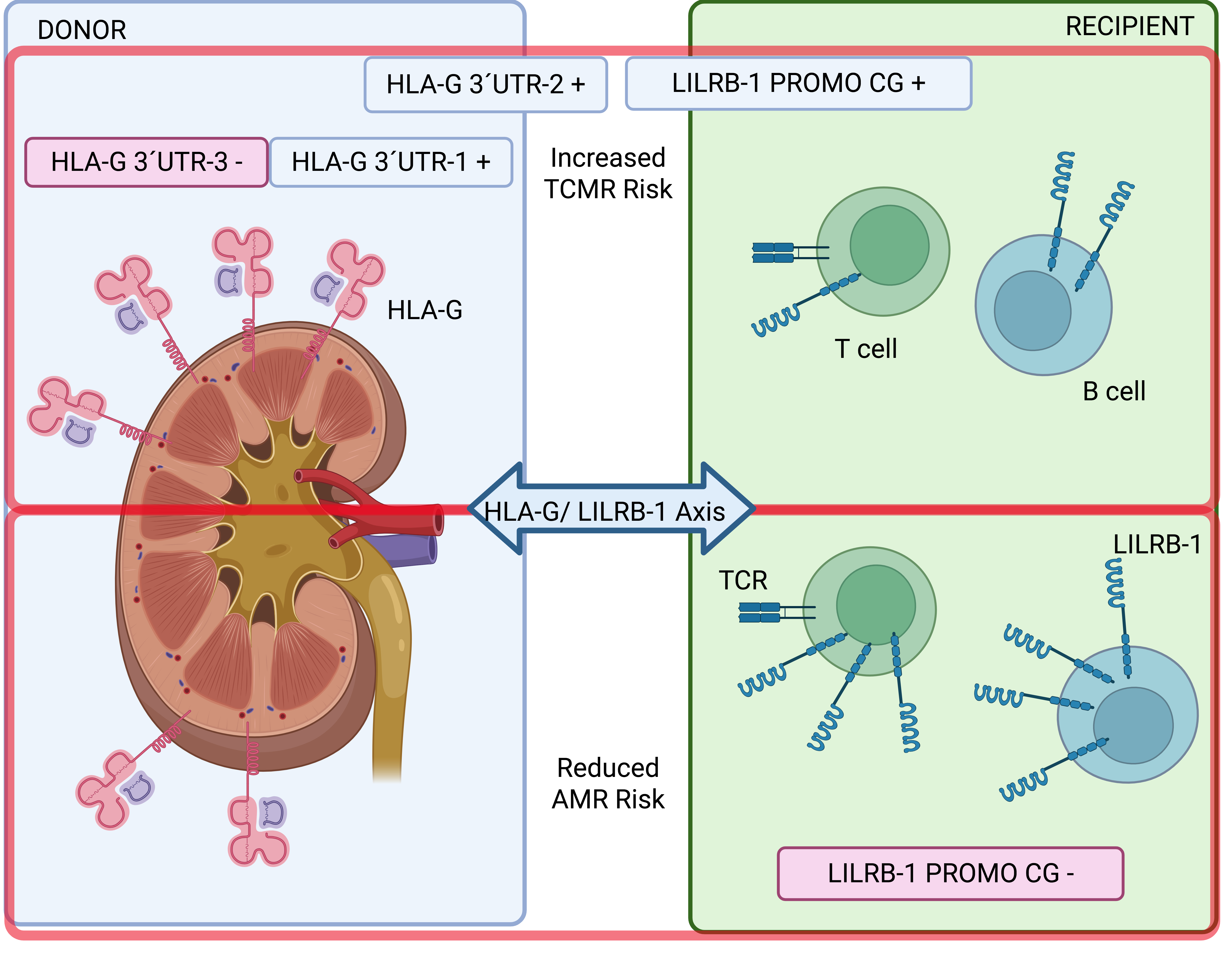

Supplement: Supplementary file 1 [file Image1.jpeg]
