# Supplementary material for: Genetic variants of the HLA-G/LILRB1 ligand-receptor axis in donors or recipients are prognostic covariates for rejection after living kidney transplantation
Source: Front Immunol. 2026 Jan 5;16:1697839. doi: 10.3389/fimmu.2025.1697839 (PMC12812672; doi:10.3389/fimmu.2025.1697839)
Supplement: Supplementary file 2 [file Table1.docx]

Additional File 1. Distribution of genotypes of HLA-G 3’UTR haplotypes in recipient (n=280) and donors (n=279).

| **Haplotype** | **Genotypes** | **Recipient** |  | **Donor** |  | ***p_a_*** | **OR** |
| --- | --- | --- | --- | --- | --- | --- | --- |
|  |  | **(N=280)** | **%** | **(N=279)** | **%** |  | **(95% CI)** |
| **UTR-1** | UTR-1/UTR-1 | 61 | 21.8 | 34 | 12.2 |  |  |
|  | UTR-1/UTR-X | 84 | 30.0 | 115 | 41.2 | 0.002 |  |
|  | UTR-X/UTR-X | 135 | 48.2 | 130 | 46.6 |  |  |
|  | UTR-1 pos | 145 | 51.8 | 149 | 53.4 | 0.7014 | 0.93 (0.67 - 1.30) |
|  | UTR-1 neg | 135 | 48.2 | 130 | 46.6 |  |  |
| **UTR-2** | UTR-2/UTR-2 | 32 | 11.4 | 25 | 9.0 |  |  |
|  | UTR-2/UTR-X | 115 | 41.1 | 118 | 42.3 | 0.6282 |  |
|  | UTR-X/UTR-X | 133 | 47.5 | 136 | 48.7 |  |  |
|  | pos | 147 | 52.5 | 143 | 51.3 | 0.7682 | 1.05 (0.76 - 1.46) |
|  | neg | 133 | 47.5 | 136 | 48.7 |  |  |
| **UTR-3** | UTR-3/UTR-3 | 5 | 1.8 | 3 | 1.1 |  |  |
|  | UTR-3/UTR-X | 37 | 13.2 | 43 | 15.4 | 0.6061 |  |
|  | UTR-X/UTR-X | 238 | 85.0 | 233 | 83.5 |  |  |
|  | pos | 42 | 15.0 | 46 | 16.5 | 0.6292 | 0.89 (0.57 - 1.42) |
|  | neg | 238 | 85.0 | 233 | 83.5 |  |  |
| **UTR-4** | UTR-4/UTR-4 | 3 | 1.1 | 8 | 2.9 |  |  |
|  | UTR-4/UTR-X | 77 | 27.5 | 68 | 24.4 | 0.2403 |  |
|  | UTR-X/UTR-X | 200 | 71.4 | 203 | 72.8 |  |  |
|  | pos | 80 | 28.6 | 76 | 27.2 | 0.7257 | 1.07 (0.73 – 1.56) |
|  | neg | 200 | 71.4 | 203 | 72.8 |  |  |
| **UTR-5** | UTR-5/UTR-5 | 0 | 0.0 | 1 | 0.4 |  |  |
|  | UTR-5/UTR-X | 13 | 4.6 | 18 | 6.5 | 0.3872 |  |
|  | UTR-X/UTR-X | 267 | 95.4 | 260 | 93.2 |  |  |
|  | pos | 13 | 4.6 | 19 | 6.8 | 0.2701 | 0.66 (0.33 – 1.39) |
|  | neg | 267 | 95.4 | 260 | 93.2 |  |  |
| **UTR-6** | UTR-6/UTR-6 | 1 | 0.4 | 0 | 0.0 |  |  |
|  | UTR-6/UTR-X | 9 | 3.2 | 9 | 3.2 | 0.6071 |  |
|  | UTR-X/UTR-X | 270 | 96.4 | 270 | 96.8 |  |  |
|  | pos | 10 | 3.6 | 9 | 3.2 | 0.8216 | 1.11 (0.44 - 2.69) |
|  | neg | 270 | 96.4 | 270 | 96.8 |  |  |
| **UTR-7** | UTR-7/UTR-7 | 2 | 0.7 | 3 | 1.1 |  |  |
|  | UTR-7/UTR-X | 30 | 10.7 | 22 | 7.9 | 0.4722 |  |
|  | UTR-X/UTR-X | 248 | 88.6 | 254 | 91.0 |  |  |
|  | pos | 32 | 11.4 | 25 | 9.0 | 0.335 | 1.31 (0.76 - 2.49) |
|  | neg | 248 | 88.6 | 254 | 91.0 |  |  |
| **UTR-13** | UTR-13/UTR-13 | 0 | 0 | 0 | 0.0 |  |  |
|  | UTR-13/UTR-X | 7 | 2.5 | 4 | 1.4 | n.a. |  |
|  | UTR-X/UTR-X | 273 | 97.5 | 275 | 98.6 |  |  |
|  | pos | 7 | 2.5 | 4 | 1.4 | 0.3641 | 1.76 (0.56 - 5.43) |
|  | neg | 273 | 97.5 | 275 | 98.6 |  |  |
| **UTR-18** | UTR-18/UTR-18 | 0 | 0 | 0 | 0.0 |  |  |
|  | UTR-18/X | 8 | 2.9 | 10 | 3.6 | n.a. |  |
|  | UTR-X/UTR-X | 272 | 97.1 | 269 | 96.4 |  |  |
|  | pos | 8 | 2.9 | 10 | 3.6 | 0.6263 | 0.79 (0.31 - 2.06) |
|  | neg | 272 | 97.1 | 269 | 96.4 |  |  |

confidence interval; HC – healthy controls; COVID-19 – coronavirus disease 2019; pos – positive; UTR – untranslated region; pa-values were calculated by GraphPad Prism using two-sided Fisher’s exact test when evaluating distribution of HLA-G 3’UTR haplotypes and Chi-Square test when assessing genotypes. alpha<0.05; OR – odds ratio; n.a. – due to low number of samples
