# Supplementary material for: Genetic variants of the HLA-G/LILRB1 ligand-receptor axis in donors or recipients are prognostic covariates for rejection after living kidney transplantation
Source: Front Immunol. 2026 Jan 5;16:1697839. doi: 10.3389/fimmu.2025.1697839 (PMC12812672; doi:10.3389/fimmu.2025.1697839)
Supplement: Supplementary file 3 [file Table2.docx]

Additional File 2. Distribution of genotypes of *HLA-G* 3’UTR-1 haplotypes in recipients with primary disease being Glomerulonephritis (GN)

| **Haplotype** | **Genotypes** | **GN yes** |  | **GN no** |  | ***p_a_*** | **OR** |
| --- | --- | --- | --- | --- | --- | --- | --- |
|  |  | **(N=280)** | **%** | **(N=280)** | **%** |  | **(95% CI)** |
| **UTR-1** | UTR-1/UTR-1 | 19 | 6.8 | 42 | 15.0 |  |  |
|  | UTR-1/UTR-X | 31 | 11.1 | 53 | 18.9 | 0.09 |  |
|  | UTR-X/UTR-X | 63 | 22.5 | 72 | 25.7 |  |  |
|  | UTR-1 pos | 50 | 17.8 | 95 | 33.9 | 0.04 | 0.6 (0.37-0.97) |
|  | UTR-1 neg | 63 | 22.5 | 72 | 25.7 |  |  |
