# Supplementary material for: Genetic variants of the HLA-G/LILRB1 ligand-receptor axis in donors or recipients are prognostic covariates for rejection after living kidney transplantation
Source: Front Immunol. 2026 Jan 5;16:1697839. doi: 10.3389/fimmu.2025.1697839 (PMC12812672; doi:10.3389/fimmu.2025.1697839)
Supplement: Supplementary file 4 [file Table3.docx]

**Additional File 3.** Sequence comparison of the most frequent HLA-G 3’UTR haplotypes.

|  | **UTR-1** | **UTR-2** | **UTR-3** | **UTR-4** | **UTR-5** | **UTR-7** | **UTR-13** | **UTR-6/ 18** |
| --- | --- | --- | --- | --- | --- | --- | --- | --- |
| +2961 | DEL | INS | DEL | DEL | INS | INS | DEL | DEL |
| +3001 | C | C | C | C | C | C | C | C |
| +3003 | T | T | T | C | T | T | T | T |
| +3010 | G | C | C | G | C | C | C | G |
| +3027 | C | C | C | C | C | A | C | C |
| +3035 | C | C | C | C | T | T | T | C |
| +3142 | C | G/C | G | C | G | G | G | C |
| +3187 | G | A | A | A | A | A | A | A |
| +3196 | C | G | C | C | C | C | C | C |
| +3227 | G | G | G | G | G | G | G | G/A |
